# Structural homogeneity and mass density of bulk metallic glasses revealed by their rough surfaces and ultra-small angle neutron scattering (USANS)

**DOI:** 10.1038/s41598-018-30333-9

**Published:** 2018-08-28

**Authors:** Man-Ho Kim, Jin-Yoo Suh, Eric Fleury, Su Gyeong Han, Kyung Tae Hong

**Affiliations:** 1 Advanced Analysis Center, Korea Institute of Science and Technology, Seoul, 02792 Republic of Korea; 2 High Temperature Energy Materials Research Center, Korea Institute of Science and Technology, Seoul, 02792 Republic of Korea; 3 LEM3, UMR CNRS 7239, Université de Lorraine, 57000 Metz, France; 4 Center for Materials Architecturing, Korea Institute of Science and Technology, Seoul, 02792 Republic of Korea

## Abstract

The ultra-small angle neutron scattering (USANS) measures the microscale structure of heterogeneity and the scattering from rough surfaces with small scattering volumes can be neglected. But this is not true in amorphous alloys. The small angle scattering from such surfaces is not negligible, regardless of scattering volume. However, we demonstrate that the unwanted rough surfaces can be utilized to determine the homogeneity and mass density of amorphous metallic glasses using the USANS and surface neutron contrast matching technique. The power law scattering of the homogeneous Cu_50_Zr_50_ amorphous alloy disappeared under the surface contrast-matched environment, a mixture of hydrogenated/deuterated ethanol having low surface tension against the metallic alloys, indicating that the scattering originated not from its internal structure but from the rough surface. This confirms the structural homogeneity not only at the atomic level but also on a larger scale of micrometer. On the other hand, the crystallized Cu_50_Zr_50_ alloy showed strong power-law scattering under the matching environment due to the structural heterogeneity inside the alloy. This technique can apply to the bulk samples when the transmission is high enough not causing multiple scattering that is easily detected with USANS and when the surface roughness is dominant source of scattering.

## Introduction

Metallic alloys with robust glass forming ability, also known as metallic glasses, consist of amorphous structure that is free of heterogeneities like crystalline phase, grain boundaries, and dislocations. The structural homogeneity of metallic glasses is widely investigated at an atomic level using X-ray diffraction and transmission electron microscopy. In only a few cases, the small angle x-ray scatterings (SAXS) and small angle neutron scatterings (SANS) have been used to measure the heterogeneities at a nanometer level^1–10^.

In addition to the well-known homogeneous nature of the amorphous metallic structure in the nanometer scale, spontaneous decomposition in larger scale, also known as phase separation, was also reported^11,12^. The molecular dynamics simulation showed density fluctuation (i.e., local structural fluctuation) on the atomic length scale in the quenched Cu_57_Zr_43_ alloy^13^. However, only limited information on the phase uniformity of metallic glasses is available in a wide range of length scales. In this context, we have questioned whether the homogeneity of metallic glasses confirmed at the atomic and nanometer levels can be sustained at a much larger scale, at least up to the micrometer scale. Ultra-small angle neutron scattering (USANS) enables us to measure the homogeneity and heterogeneity up to tens of micrometers. Due to the strong penetration power of neutrons into most alloys with the interaction of a neutron to nucleus, USANS and SANS are excellent tools to investigate the structure of thick samples over hundreds of micrometers or even thicker samples when the transmission is high enough not to cause multiple scattering, while SAXS may require the specimen to be thinned down to a few microns in materials with high atomic number due to the shallow penetration capability caused by the interaction of the electromagnetic waves with electrons that have a higher number density than nucleus.

Bulk metallic ribbons have inherent rough surface of which surface topology has been known to affect properties such as corrosion, friction, wear, and plastic deformation^14,15^. For bulk samples in most alloys, the surface roughness can be less important in USANS and SANS measurements due to the smaller surface-to-volume ratio than SAXS^16^. However, in amorphous materials like BMGs, scattering from the surface cannot be neglected even though the scattering volume is considerably less than that of bulk samples, since the internal homogeneous (i.e., no density and composition fluctuation) phase must not have any structures that cause scattering. In fact, Roth demonstrated that the Porod scattering (i.e., the scattering intensity decreases with a power of −4 against Q, representing a sharp interface of a structure) from SANS originated from the surface irregularities and oxide layers of pure polycrystalline metals^17^. Rodmacq *et al*. showed the influence of surface conditions on the SANS profile of Pd_80_Si_20_ alloys by measuring specimens under different surface treatments: quenched, polished, and chemically etched^18,19^. Therefore, it is likely that the rough surface causes the non-trivial surface scatterings which could lead to misinterpretation of SANS and USANS data as the homogeneous amorphous alloys have inner heterogeneity.

Thus, the surface roughness hinders us from determining the structural homogeneity of metallic glass structure. In addition, the evaluation of the mass density of an amorphous metallic glasses with the rough surface is another challenging task. For example, in the well-known Archimedes method, entrapped air bubbles can cause lower density when high surface tension liquids are used^20,21^, or by the closed pores that liquids cannot be accessible. Also, if any internal heterogeneities are present, the measured density can be misunderstood as the density of the sample in a pure homogeneous state, unless homogeneities or heterogeneities are confirmed with other density measurement methods. In this study, however, we will show how the rough surface, which is not desired in most characterization methods, can be applied to determine the microscale homogeneity and the mass density of BMG alloys simultaneously using the unique contrast-matched USANS technique. For this purpose, USANS is more useful than SANS because only USANS can properly evaluate the typical micron-sized roughness. Also, USANS is less sensitive to incoherent backgrounds than SANS.

## Surface neutron contract-matching (SNCM) method

**Figure 1:**
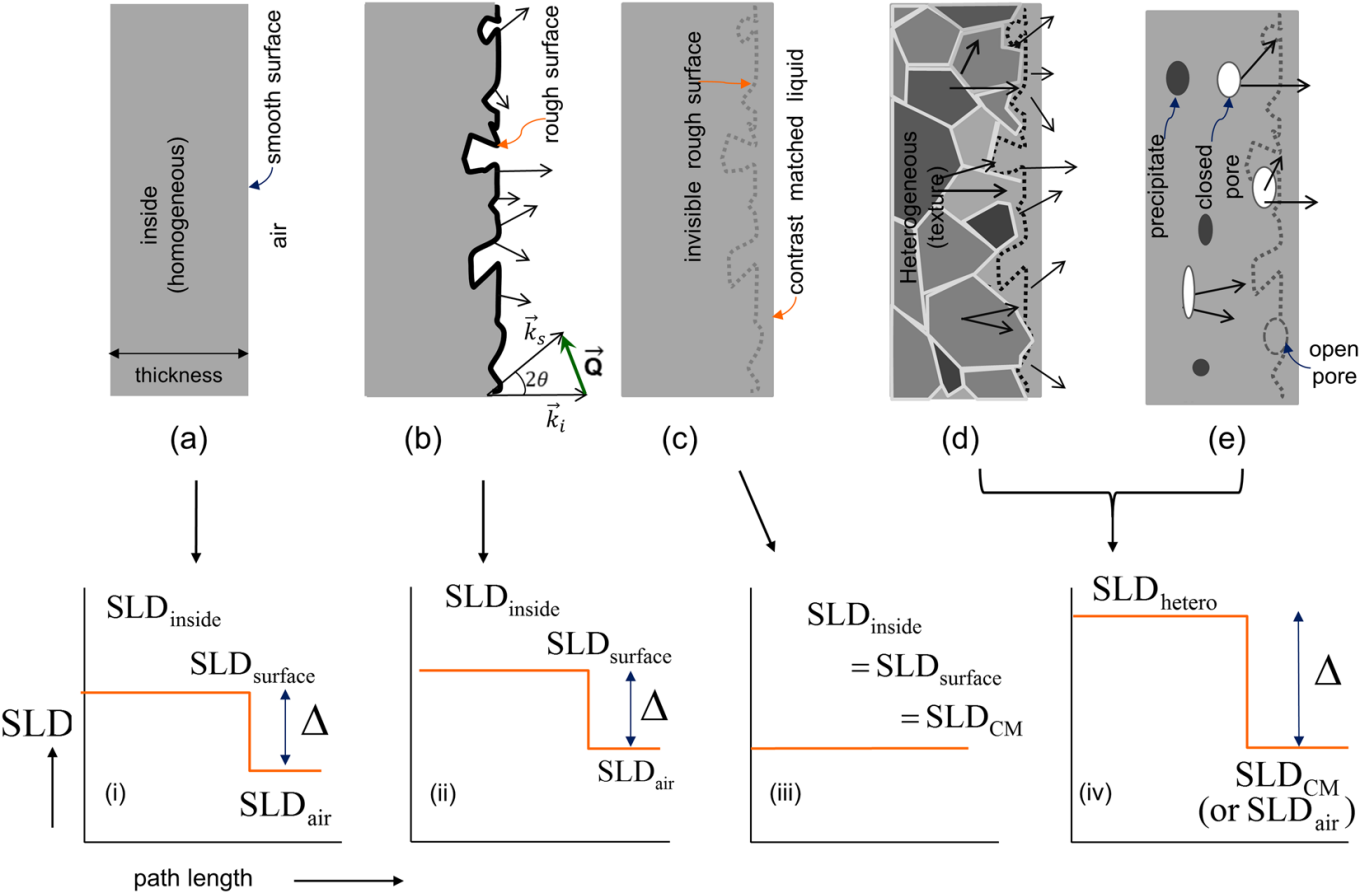
Schematics of ultra-small and small angle scatterings at various sample environments of amorphous alloy: (**a**) homogeneous BMGs with smooth surface, (**b**) homogeneous BMGs with rough surface, (**c**) homogeneous BMGs with rough surface at surface neutron contrast-matched (SNCM) environment. (**d**) crystallized (i.e., heterogeneous) BMGs with rough surface, and (**e**) BMGs with precipitates, pores, and/or rough surface. Corresponding SLD profiles with path length (i.e., sample thickness) are displayed in (i–iv). SLD_hetero_ indicates heterogeneity from precipitates, rough surfaces, or/and voids. The symbol Δ represents the difference between the SLDs. No scatterings are expected in (**a**)-(i) condition because there are no scatterers (i.e. ϕ(1−ϕ) = 0) even with a difference in SLD between surface and air and in (**c**)-(iii) condition because of no contrast (i.e., Δ = 0). Sample thickness and roughness are not scaled. The samples have two surfaces that contribute to the forward scatterings, but for clarity, note that the schematics show only one-sided surfaces. $\overrightarrow{{{\boldsymbol{k}}}_{{\boldsymbol{i}}}}$ and $\overrightarrow{{{\boldsymbol{k}}}_{{\boldsymbol{s}}}}$ are the incident and scattered wave vectors, respectively. $\overrightarrow{{\boldsymbol{Q}}}$ is the scattering vector.

Expected small angle scatterings from an amorphous alloy can be schematically shown in Fig. 1. Small angle scattering is registered on the detector when scattering materials have a contrast (Δ*SLD*)^2^ defined as the squared difference of the coherent scattering length densities (SLD)^22,23^ and/or volume fraction ϕ(1 − ϕ) between domains (or elements), i and j:

1 $$\frac{d{\rm{\Sigma }}}{d{\rm{\Omega }}}({\rm{Q}})\sim {\rm{P}}({\rm{Q}}){\rm{S}}({\rm{Q}}){{\rm{\varphi }}}_{i}(1-{{\rm{\varphi }}}_{i}){(SL{D}_{i}-SL{D}_{j})}^{2}$$

where the macroscopic scattering cross-section (cross section per unit volume, cm^−1^), *d*Σ(*Q*)/*dQ*, is a function of the scattering vector, Q = (4π/λ)sin(θ), defined with the neutron wavelength λ and the scattering angle 2θ. P(Q) and S(Q) is a form factor that represents a shape of scattering object (i.e., scatterer) and a structure factor that does an inter-correlation distance, respectively.

For the homogeneous phase (i.e., single phase) as shown in Fig. 1(a), no small angle scattering must be observed since Eq. (1) becomes equal to zero since there are no scatterers (i.e., ϕ(1 − ϕ) = 0) due to internal homogeneity and smooth surface even with a difference in SLD between surface and air (see also Table 1). Unlike our expectation, the preliminary USANS and SANS results of Cu_50_Zr_50_ (see Fig. S1 of supplementary information) and literature^5,17–19^ show strong power-law scattering under air environment, which may indicate significant heterogeneities in the metallic glasses. However, since the homogeneous BMG alloys have no scattering regardless of scattering volume (i.e, thin or bulk samples), the rough surface (Fig. 1(b)) could be a reason for the power law or fractal-like scattering. In this case, the origin of the power law scattering may be due to the contrast (SLD_surface_−SLD_air_)^2^ between the rough surface SLD_surface_ and the air environment SLD_air_ (as shown in Fig. 1(ii)), even with the inner homogeneity of the metallic glass. When the rough surface is contrast-matched (CM) to the neutron SLD of the homogeneous phase to make SLD_inside_ = SLD_surface_ = SLD_CM_ (i.e., the air environment is replaced with a surface neutron contrast-matched (SNCM) environment by filling the surface furrows with a matching liquid which masks the rough surface from neutrons (Fig. 1(c)), the scattering from the rough surface should not be observed because of no contrast as shown schematically in Fig. 1(iii). It is shown that the scattering from the rough surface or open networked-pores can be suppressed by the neutron contrast-matching technique^19,22,24^.

**Table 1:** Summary of scattering origin based on Fig. 1 and Eq. (1).

	(a)	(b)	(c)	(d) and (e)
(Δ*SLD*)^2^	>0	>0	0 (contrast matched)	>0
ϕ(1 − ϕ)	0 (no scatterers)	>0	>0	>0
$\frac{d{\Sigma }({Q})}{d{\Omega }}$	0 (no scattering)	>0	0 (no scattering)	>0

When the sample has heterogeneities, such as polycrystals with boundaries (Fig. 1(d)) and dispersion of particles or voids (Fig. 1(e)) in a matrix, USANS and SANS scatterings from such heterogeneities occur due to a contrast mismatching, i.e.,$({{\rm{SLD}}}_{{\rm{hetero}}}\ne {{\rm{SLD}}}_{{\rm{CM}}})$, with the CM liquid matched to the homogeneous phase (Fig. 1(iv)). In this case, the surface contrast may be selectively matched to only one of the phases in a system comprising multi heterogeneous domains (Fig. 1(d) and (e)), which may enable us to measure the other phases or pores.

In the process of finding the optimum liquid composition that matches the SLD of a constituent phase, the mass density of the constituent phase could be estimated. The procedure for density estimation will be presented in detail in the later part of this paper. Overall, the schematics in Fig. 1 will be demonstrated and used to determine the microscale homogeneity and the mass density of BMG alloys.

## Results and Discussion

### Homogeneity in atomic level

**Figure 2:**
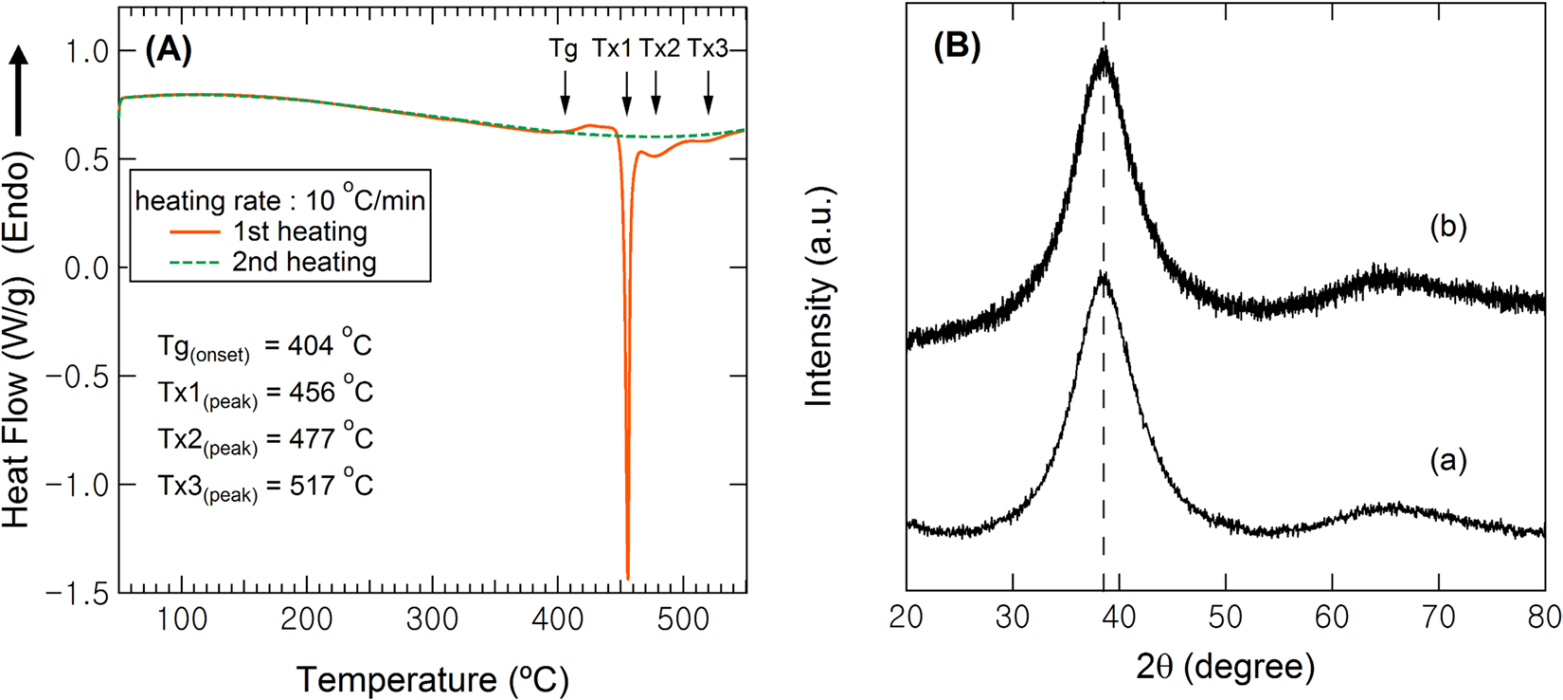
(**A**) DSC and (**B**) XRD scans to confirm homogeneity in the atomic level of Cu50Zr50. (**A**) DSC traces of as-received Cu50Zr50 (the 1st run, orange solid line) followed by the 2nd run (green dotted line), (**B**) X-ray diffraction spectra of (a) as-cast and (b) sub-T_g_ annealed Cu_50_Zr_50_ at 400 °C for 1 hr. Note that no oxide layer peak was observed in the polished sample (b).

Before determining the homogeneity and the mass density using USANS, the structure of Cu_50_Zr_50_ metallic glass samples in the form of ribbons was checked by DSC thermal analysis and X-ray diffraction (Fig. 2).

The DSC thermal scan was carried out twice to confirm the complete crystallization after the first scan up to 550 °C; upon cooling and reheating of the same specimen for the second run, the heat flow curve (dotted green line in Fig. 2(A)) was almost constant suggesting complete crystallization of the amorphous phase during the first heating (orange solid line). For the XRD spectra (shown in Fig. 2(B)), the two broad amorphous halos and full width at half maxima (FWHM) of both the as-cast and sub-T_g_ annealed samples were identical within a lab-scale XRD error range indicating the same atomic number density and no noticeable change in correlation distances between atoms (i.e., identical amorphous structures in both the as-cast and sub-T_g_ annealed Cu_50_Zr_50_). Results of the X-ray and DSC analyses confirm that Cu and Zr elements in the as-cast and sub-T_g_ annealed Cu_50_Zr_50_ are homogeneously mixed and do not form any crystallite at the atomic level (in the available range of lab-scale XRD). The USANS measurements were undertaken to investigate whether the homogeneity in the amorphous phase is limited at the atomic level or is extended to larger scale, i.e., micrometers.

### Determination of homogeneity and heterogeneity in the micron scale with SNCM technique

**Figure 3:**
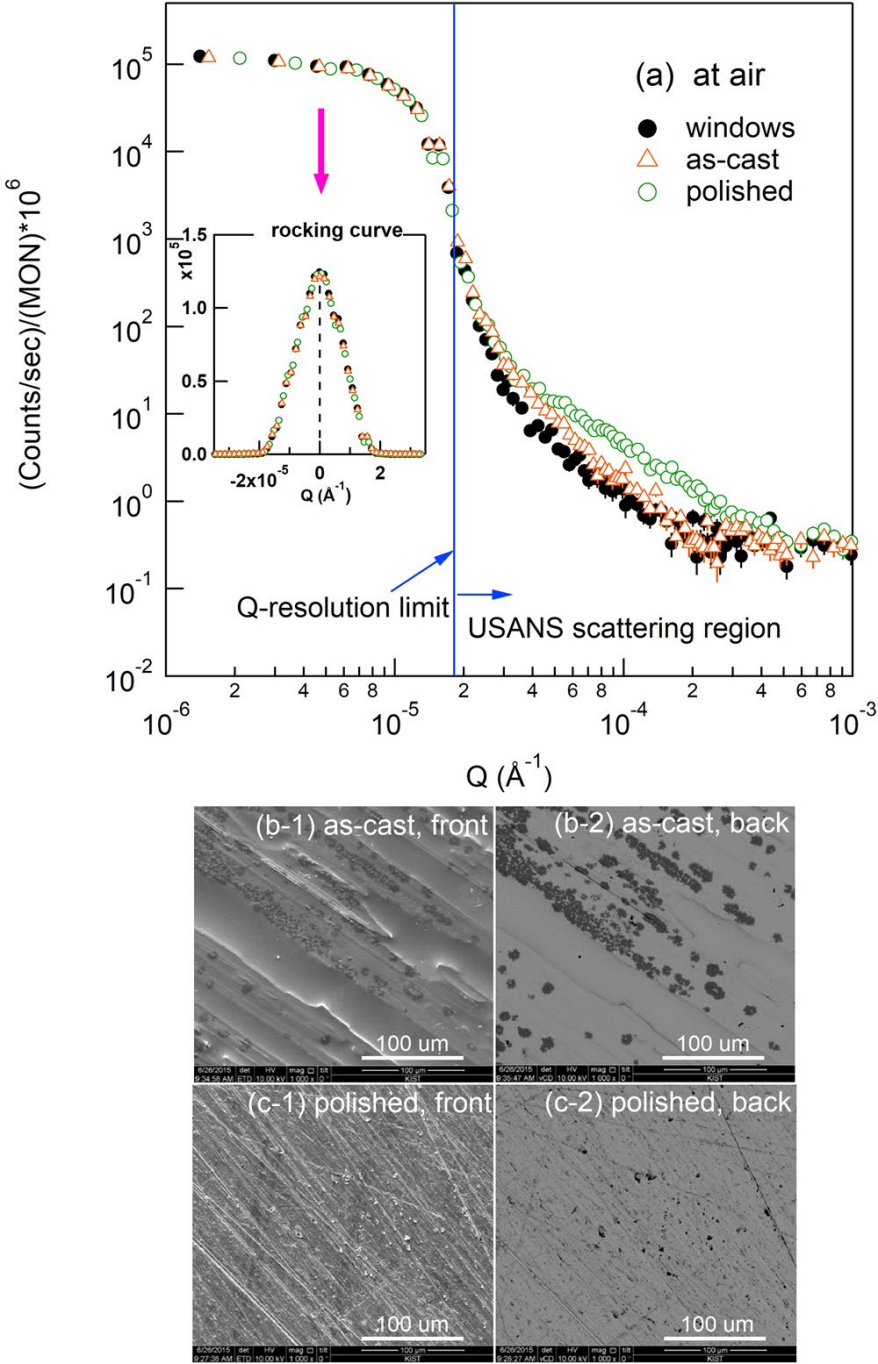
(**a**) Ultra-small angle neutron scatterings of quartz windows of sample container (closed circle in black color), as-cast (open triangles in orange color), and polished Cu_50_Zr_50_ (open circles in green color) measured at air environment. SEM images of (**b**) as-cast and (**c**) polished sample with additional numbering ‘-1’ for front face (air-side during melt-spinning) and ‘-2’ for back face (roll-side during melt-spinning) are also shown. USANS resolution limit and scattering region are indicated in (**a**). Inset shows the full rocking curves on a linear scale while the halves of the rocking curves are shown on a double logarithm scale. The scale bar in the SEM image indicates 100 um.

Before investigating the homogeneity at the micrometer level, the USANS profiles of the Cu_50_Zr_50_ ribbons without surface polishing (open orange triangle) and with polishing (open green circle) by means of the sandpapers (600, 1000, and finally 2000 grade in order) were compared with air scattering (closed circle) in Fig. 3(a).

Scatterings from both samples are stronger than the window scattering from the empty sample container, indicating the presence of effective scatterers (i.e., heterogeneity in the structure) which must not be observed in the homogeneous BMG as shown in Fig. 1(a) and Table 1. The polished sample (open circles in green color, Fig. 3(a)) shows higher intensity around Q ~ 2 × 10^−4^ ~3 × 10^−3^ Å^−1^ than the as-cast sample (open triangles in orange color, Fig. 3(a)), suggesting a greater number of scatterers in the surface-treated (polished) sample. Note also that the scattering intensities and shapes of the rocking curves in the Q-resolution limit with/without the Cu_50_Zr_50_ samples are almost identical (i.e., no attenuation of intensity and no beam broadening at all as shown in inset), resulting in high transmission around 92% (+/−2%) and ensuring no multiple scattering (see Method section later regarding details of multiple scattering).

The SEM image shows sharper scratches with significantly higher number density for the surface-treated sample [image (c-1) and (c-2) of Fig. 3] than on the surface of the as-cast sample [image (b-1) and (b-2) of Fig. 3], suggesting that the surface scratches (i.e. rough surface) could be the origin of the USANS scattering. However, in the case of metallic glass ribbons having both macro-heterogeneity and rough surface, it would not be clear whether the power law scattering results from the large heterogeneity (i.e., much larger than atomic scale) inside the sample or from the rough surface [Fig. 1(d) and (e)]. To identify the origin of the scattering and to determine the homogeneity in much larger size scale than atomic level, the USANS measurements were performed at different sample environments corresponding to each schematic diagrams of Fig. 1 for the homogeneous Cu_50_Zr_50_ alloy.

**Figure 4:**
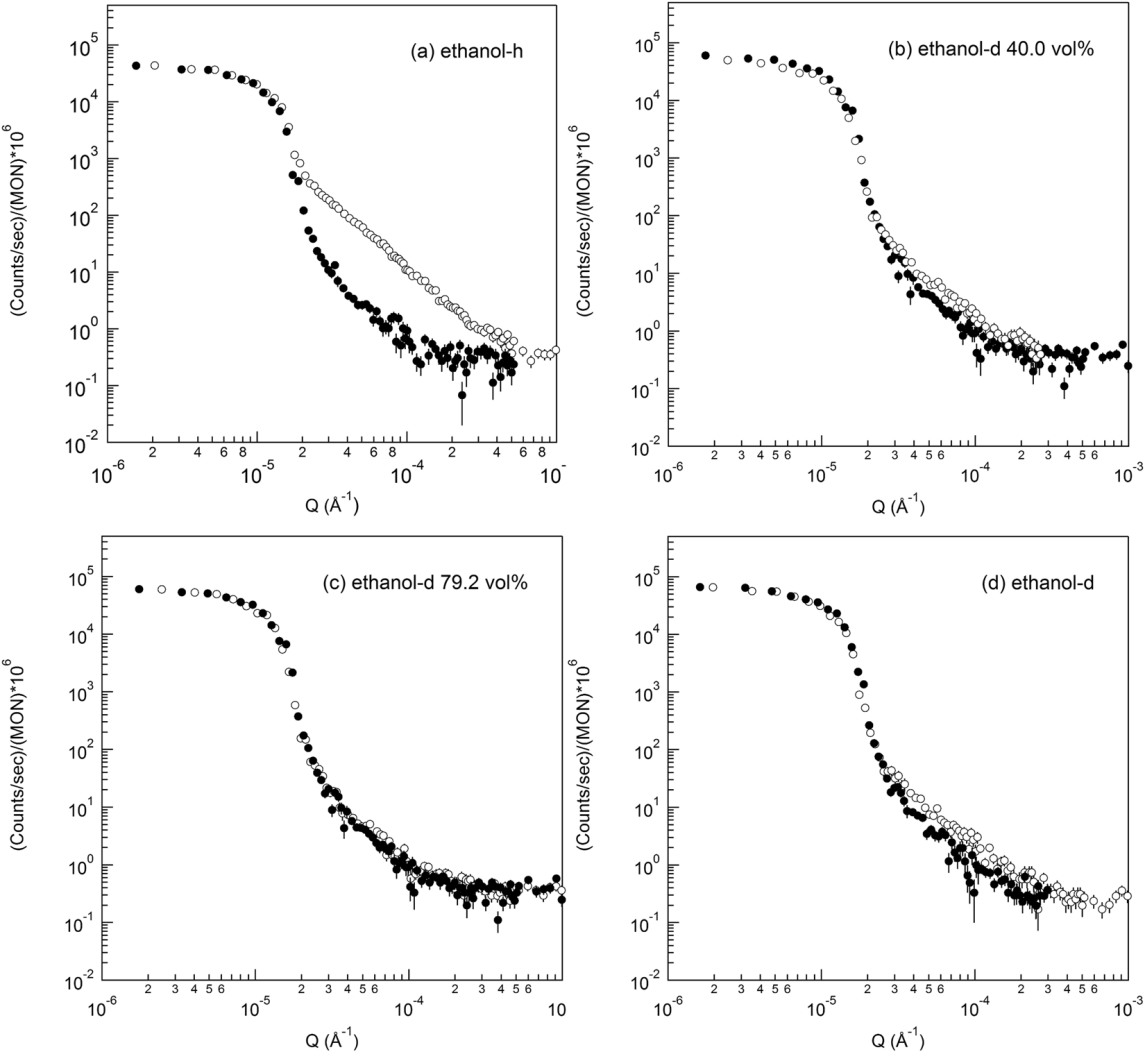
Ultra-small angle neutron scatterings of the polished Cu50Zr50 (open) immersed in different buffers: (**a**) pure ethanol-h, (**b**) mixture of ethanol-h/ethanol-d, 60/40 vol./vol., (**c**) mixture of ethanol-h/ethanol-d, 20.8/79.2 vol./vol., and (**d**) pure ethanol-d. Scatterings from the buffers are shown as closed symbols.

The sample was placed in several buffers from 100% hydrogenated ethanol (C_2_H_5_OH, ethanol-h, Fig. 4(a)), ethanol-h/ethanol-d of 60/40 vol./vol. (Fig. 4(b)), 29.8/79.2 vol./vol. (Fig. 4(c)), and 100% deuterated ethanol (C_2_D_5_OD, ethanol-d, Fig. 4(d). The samples (open circles) show different scattering intensities from the buffers (closed circles) due to a difference in contrast (i.e.,(Δ*SLD*)^2^) between the sample and liquid buffer as shown in Tables 2 and 3.

**Figure 5:**
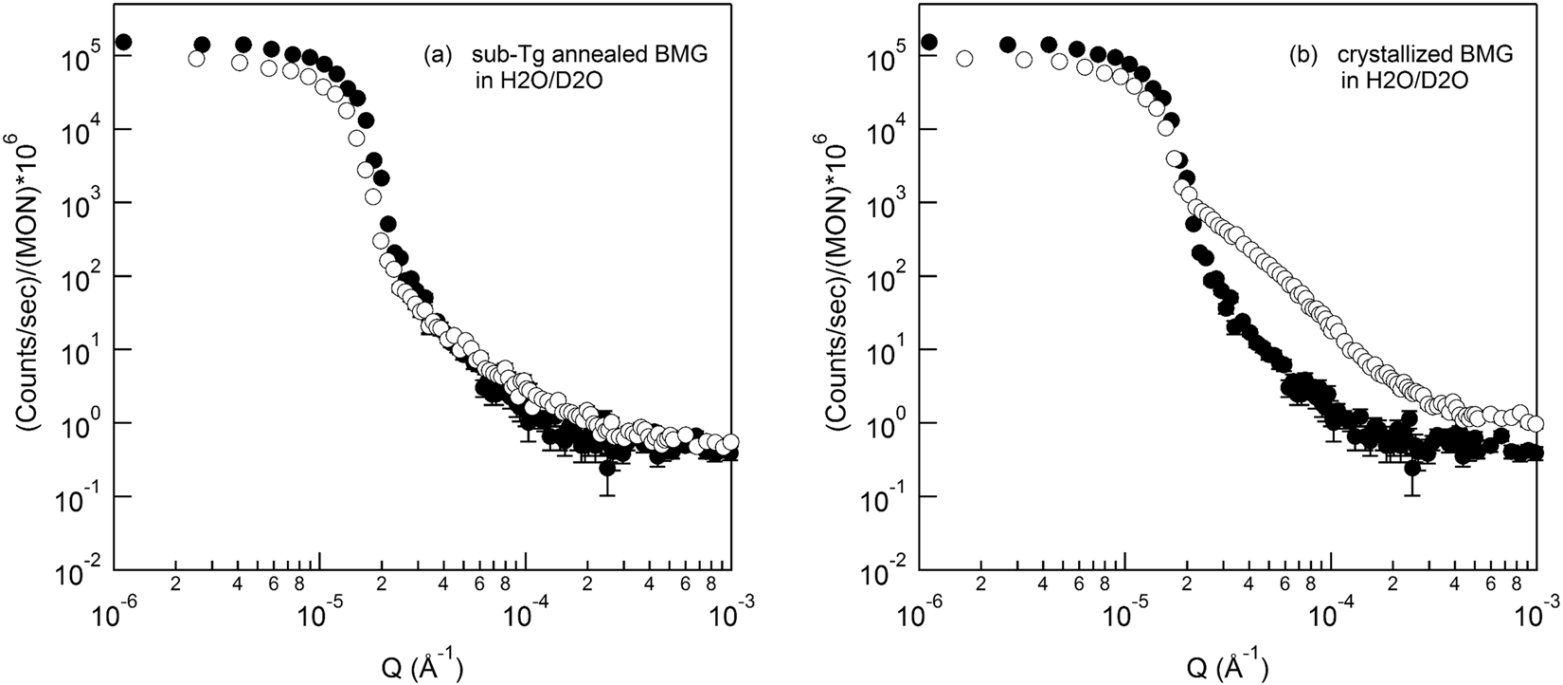
Ultra-small angle neutron scatterings of (**a**) homogeneous and (**b**) heterogeneous Cu50Zr50 alloy measured at the apparently contrast-matched 30/70 H2O/D2O vol./vol. buffer. Closed and open symbols are from the buffer only and the alloy sample immersed in the buffer, respectively.

**Table 2:** Neutron bound coherent scattering lengths, b, of elements for calculating SLD of ethanol mixture and Cu50Zr50^a**)**^.

elements	b × 10^13^ (cm)^b^
H	−3.739
D	6.671
C	6.646
O	5.803
Cu	7.718
Zr	7.160
Cu_50_Zr_50_	7.439

^a^reference^30^

^b^Read the numbers in the column as −3.739 × 10^−13^, 6.671 × 10^−13^, and so on.

**Table 3:** Density and SLD of Cu50Zr50, water, ethanol, and contrast matched ratio at 25 °C.

	Mw(g/mole)	density (g/cm^3^)	SLD × 10^−10^ (cm^−2^)^a^	ΔSLD^2^ × 10^−20^ (cm^−4^)^b^
Cu_50_Zr_50_	77.3850	7.3(1) ± 0.2(4)^c^	4.2296	
H_2_O	18.0148	1.0000	−0.55993	23.0508
D_2_O	20.0272	1.1070	6.3988	17.9878
ethanol-h	46.0684	0.7850	−0.3426	21.0131
ethanol-d	52.1056	0.8910	6.1123	3.49241
ethanol-h/ethanol-d (29.1/70.9 vol./vol.)^d^	50.3488	0.8602	4.2296	

^a^Read the numbers in the column as −0.55993 × 10^10^, 6.3988 × 10^10^ and so on.

^b^Read the numbers in the column as 23.0508 × 10^20^, 17.9878 × 10^20^ and so on.

^c^Estimated from this study using the squared invariant. Also, from the squared zero-angle scattering method, we obtained the density of ρ_Cu50Zr50_ = 7.2(4) ± 0.2(3) (see main text for details)

^d^from the matching ratio, 70.9(9) vol. % of ethanol-d from SNCM plot of squared root invariants

The sample immersed in the 100% hydrogenated ethanol-h (Fig. 4(a)), shows strong scattering due to the large contrast, while the same specimen immersed in the 100% deuterated ethanol-d shows weak scattering (Fig. 4(d)). In the mixture of ethanol-h/ethanol-d, 20.8/79.2 vol./vol. ratio (Fig. 4(c)), the scattering (open circles) from the sample almost superimposes to the scattering (closed circles) from the buffer liquid of 79.2% ethanol-d, confirming that the SLD of the buffer liquid is close to that of the homogeneous Cu_50_Zr_50_ alloy. The estimated density of Cu_50_Zr_50_ alloy from this ratio is approximately 8.23 g/cm^3^ that is about 11–14% higher than the known densities, 7.23 ~ 7.41 g/cm^3^, in the literature^25–28^. The exact matching ratio and density determination will be shown from a SLD matching plot later in the section of mass density estimation. Figure 4(c) indicates that the USANS scattering of the BMG is not from the internal heterogeneous structure but mostly from the rough surface (i.e., no scattering from the inside as shown in Fig. 1(c) and 1(iii)).

**Figure 6:**
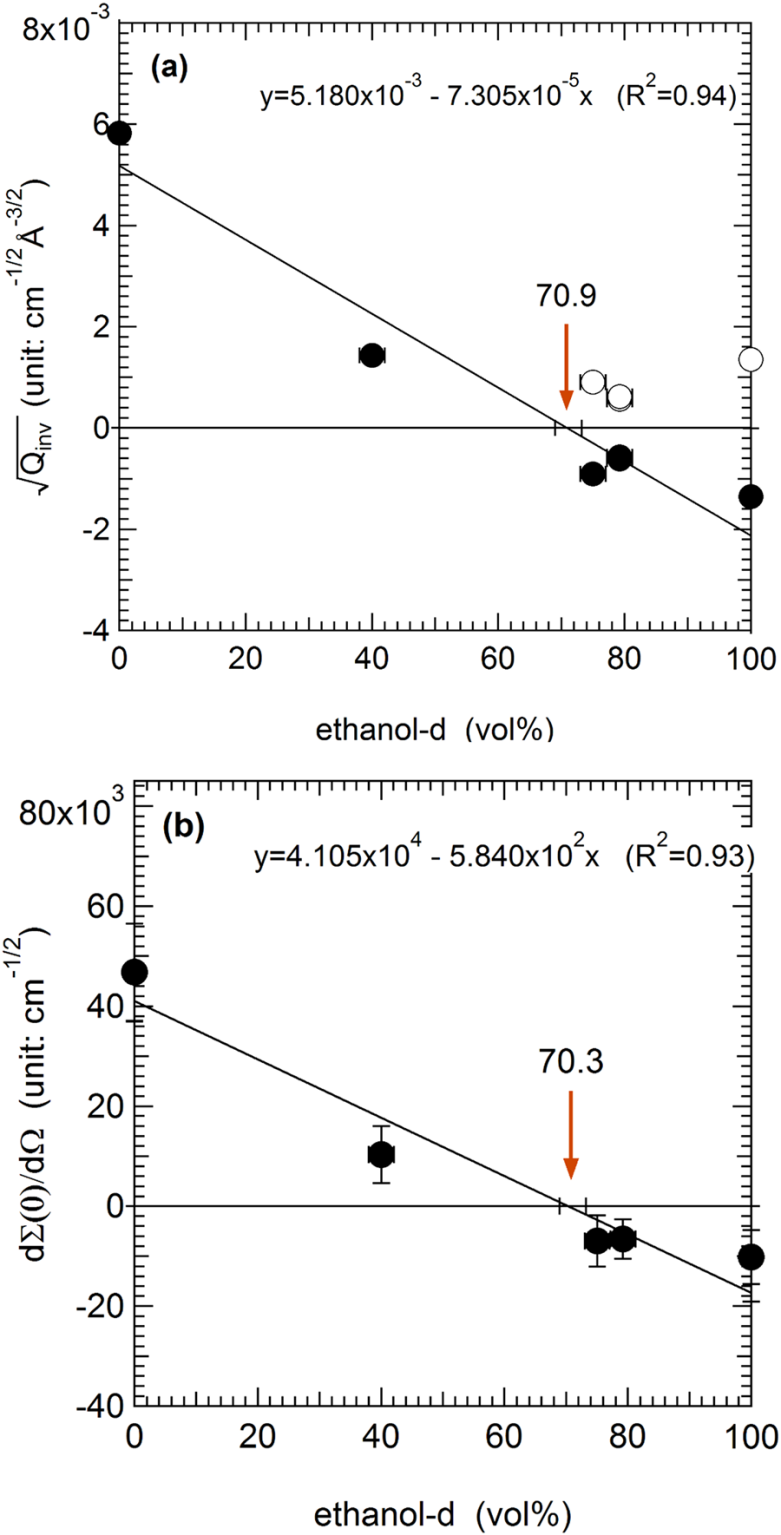
SNCM (Surface neutron contrast-matching) plot of (**a**) squared root invariants and (**b**) squared root of extrapolated zero angle scattering against ethanol-d content in the mixture of ethanol-h/ethanol-d. For (**a**), open circles are the squared root of extrapolated zero angle scattering against ethanol-d content in the mixture of ethanol-h/ethanol-d. For (**a**), open circles are the squared root invariants measured with five ethanol-d contents (0, 40.0, 75.0, 79.2, and 100%). The negative values (closed circle) for 75.0, 79.2, and 100**%** were used to find the ethanol mixing ratio at which the contrast is zero by linearly fitting the squared root invariants as a function of ethanol-d content. Surface contrast matching point is in the range of 68.9% ~73.2**%** (indicated as error bar on x axis) and a corresponding mass density is approximately 7.3(1) ± 0.2(4) g/cm^3^ from the matching ratio 70.9(9) ± 2.1(3) vol. % of ethanol-d (indicated as an orange arrow). The errors in vertical direction is within the symbols [see Appendix]. (**b**) Surface contrast matching point from the zero angle scattering is in the range of 68.1(9) % ~72.4(1) %) and a corresponding mass density is approximately 7.2(4) ± 0.2(3) g/cm^3^ from the matching point 70.2(9) ± 2.1 vol. % of ethanol-d (indicated as an arrow). The vertical error bar was estimated from the count statistics [see Appendix]. The horizontal error bars are assumed to be 3% in both (**a**) and (**b**) methods.

The USANS scattering phenomena measured with the ethanol-h/ethanol-d mixtures with low surface tension were cross-checked with the H_2_O and D_2_O mixtures with high surface tension in the same manner as that of the ethanol mixture. Figure 5(a) shows that the USANS scattering (open circles) of the Cu_50_Zr_50_ immersed in the mixture of 30/70 H_2_O/D_2_O vol./vol. almost superimposes to the empty scattering (buffer liquid only, closed circles) within experimental error, confirming that the SLD of the mixture is close to that of the homogeneous Cu_50_Zr_50_ alloy. However, when the USANS of the Cu_50_Zr_50_ crystallized at 500 °C (see Fig. S2 for XRD) was measured with the same H_2_O/D_2_O mixture, strong power law scattering was observed (open circle in Fig. 5(b)) due to the large differences in the contrast (i.e., due to large mismatching in SLDs between crystalline domains of the crystallized Cu_50_Zr_50_ alloy and 30/70 H_2_O/D_2_O vol./vol. mixture), indicating the existence of inner heterogeneity as schematically shown in Fig. 1(d) and 1(iv). Figures 4 and 5 demonstrate that the homogeneity and heterogeneity (schematically illustrated in Fig. 1) of the Cu_50_Zr_50_ alloy in the large scale can be determined from the rough surface at the SNCM condition.

### Mass density estimation

The rough surface can be further applied to determine the mass density of homogeneous metallic glasses together with the SNCM technique. Two methods were used in this study: the invariant method and the zero-angle scattering method. The invariant, Q_inv_, defined as:

2 $${Q}_{inv}=2{\pi }^{2}\varphi (1-\varphi ){({\rm{\Delta }}SLD)}^{2}$$

is used^23^. In Eq. (2), the *Q*_*inv*_ depends only on the contrast term, (Δ*SLD*)^2^, in a two-phase system that can be controlled by tuning the sample environment as shown in the CM experiments, since the volume fraction ($\varphi$) of the empty space in the rough surface and the rest, (1 − $\varphi$), composed of the Cu_50_Zr_50_ materials are constant. *Q*_*inv*_ is obtained from the area of the invariant plot, $(d{\rm{\Sigma }}(Q)/d{\rm{\Omega }})\cdot {Q}^{2}$ vs.*Q*, in the desmeared scattering profile:

3 $${Q}_{inv}={\int }_{{Q}_{{\rm{\min }}}}^{{Q}_{{\rm{\max }}}}\frac{d{\rm{\Sigma }}}{d{\rm{\Omega }}}(Q)\cdot {Q}^{2}dQ$$

where ${Q}_{{\rm{\min }}}$ and ${Q}_{{\rm{\max }}}$ are the minimum and maximum Q, respectively.

The quadratic contrast term can be linearized by taking $\sqrt{{Q}_{inv}}$ (≈|ΔSLD|) and by reflecting one side of $\sqrt{{Q}_{inv}}$ values about the abscissa in the plot of $\sqrt{{Q}_{inv}}$ versus deuterated liquid amount in the H/D mixture^22,29^. The positive values (open circles) of $\sqrt{{Q}_{inv}}$ for 75.0, 79.2, and 100% ethanol-d were reflected to the negative side (closed circles) with respect to the x-axis as shown in Fig. 6(a). The error bars in vertical direction calculated using Eq. (B5) are not shown because they are screened by the symbols. [The propagation of uncertainty is derived in the Appendix]. If the system is a two-phase alloy (i.e., consisting of the homogeneous phase of metallic glass and the empty space in rough surface), the data must align linearly and the exact surface neutron CM ratio is determined from the x-intercept that represents ΔSLD = 0.

Figure 6(a) shows the linearity of $\sqrt{{Q}_{inv}}$ with the deuterated ethanol, which means that the Cu_50_Zr_50_ phase is homogeneous at the micron scale. The results confirm that the well-known homogeneity at the atomic level is extended to the micron size in scale. From the matching ratio, 29.1/70.9 vol./vol., of ethanol-h/ethanol-d, the mass density *ρ*_*CM*_ = 0.860(2)g/cm^3^ of the matching mixture is obtained from ${\rho }_{CM}={\varphi }_{{\rm{H}}}{\rho }_{{\rm{H}}}\,+\,{\varphi }_{{\rm{D}}}{\rho }_{D}$, where the subscripts H and D represent the hydrogenated and the deuterated liquid, respectively. Using the values of known densities and SLDs given from Tables 2 and 3, the SLD of the contrast-matched ethanol mixture is SLD_CM_ = 4.2296 × 10^−10^cm^−2^. Figure 1(c) and 1(iii) show schematic representations of the contrast-matched environment and Fig. 6 shows the corresponding experimental results with the SLD_CM_ of the ethanol mixture.

Since the SLD of Cu_50_Zr_50_ must be the same as that of the matched mixture at the SNCM condition (i.e., SLD_Cu50Zr50_ = SLD_CM_), the mass density of homogeneous BMG is determined using the following relation:

4 $${\rho }_{BMG}=\frac{SL{D}_{CM}{(\sum _{i}^{n}{n}_{i}{M}_{i})}_{BMG}}{{N}_{A}{(\sum _{i}^{n}{n}_{i}{b}_{i})}_{BMG}}$$

where ${(\sum _{i}^{n}{n}_{i}{M}_{i})}_{BMG}$ and ${(\sum _{i}^{n}{n}_{i}{b}_{i})}_{BMG}$ are the molecular weight and the scattering length of BMG consisting of atomic species i with the number (*n*_*i*_) of atoms i, atomic weight (*M*_*i*_) and scattering length (*b*_*i*_), and N_A_ is Avogadro number. The mass density of Cu_50_Zr_50_ using Eq. (4) and the values listed in Tables 2 and 3 is estimated as ρ_Cu50Zr50_ = 7.3(1) ± 0.2(4) g/cm^3^. The mass density, from the rough surface and the USANS surface neutron contrast-matched) method agrees with the density of the literature, 7.25^25^, 7.2^3 ^^26^, 7.39^27^, and 7.41 g/cm^3 ^^28^. This invariant method was further cross-checked with a plot of the squared root of the zero-angle scattering (Fig. 6(b)), since the macroscopic scattering cross-section is also proportional to the contrast term (see Eq. (1)) like the invariant^29^. The vertical error bar in Fig. 6(b) is expressed in Eq. (A12). The USANS resolution limit, Q = 2 × 10^−5^Å^−1^, was used as the zero-angle, *d*Σ(*Q* → 0)/*d*Ω, for convenience since the scattering cross-section at Q = 0 cannot be determined in the power law scattering. It should also be noted that other low Q limits can be used and it does not affect the match point. The squared root zero-angle scattering method shows a mass density of approximately ρ_Cu50Zr50_ = 7.2(4) ± 0.2(3) g/cm^3^, which is identical with the invariant method with an approximately 1% difference.

## Conclusion

The results demonstrate that the rough surface of amorphous metallic materials, which is a result of manufacturing process or intended purpose, can be used to determine the homogeneity and mass density of the bulk metallic glasses when the surfaces are sufficiently rough. The power-law scattering of amorphous Cu_50_Zr_50_ melt-spun ribbons with rough surface in the USANS measurements disappeared at the SNCM (surface neutron contrast-matched) condition which can be optimized by finding a proper composition of ethanol-h/ethanol-d mixture as well as H_2_O/D_2_O mixture within the experimental error. However, the crystallized ribbons showed strong power-law scattering with the matching mixture suggesting that the inside of the alloy is heterogeneous. This confirms that the small angle scattering originates from the rough surface and that, at the same time, the BMG consists of the homogeneous single phase. The compositional homogeneity of the amorphous metals down to the atomic level turned out to hold over an even a wider range of scale: nanometer to micrometer, over 4 orders.

It was also demonstrated that the rough surface can be used to determine the mass density of a homogeneous amorphous metallic ribbon, Cu_50_Zr_50_.

Therefore, by utilizing USANS, which has much higher Q resolution and is less sensitive in incoherent scattering than the typical SANS, the SNCM technique can be an alternative characterization method for determining homogeneity of BMG in a larger scale (i.e., micron level) than the atomic level and can be used for estimating the mass density of homogeneous BMG with a rough surface. Our method can also be applied to much thicker BMG alloys due to their high transparency to neutrons

## Methods

### Sample preparation

Cu_50_Zr_50_ ribbons of ~80 μm in thickness and ~75 mm in width were produced by the melt-spinning method at Eco-FM Company, Incheon, Korea. The ribbons were isothermally annealed under a vacuum environment at a temperature of 400 °C for 1 hour to homogenize the as-cast structure by equilibrating at a temperature slightly under the glass transition point (T_g_ ~ 404 °C). The metallic glass ribbons were also crystallized at 500 °C above a crystallization temperature (T_x1_ = 456 °C, see Fig. 2(A)) under a vacuum environment. To remove oxide layers that can evolve on the sample surface during heat treatment, the samples were polished using 600, 1000, and 2000 grade sandpapers.

### Thermal Analysis and X-ray Diffraction

Thermal analysis was carried out using a differential scanning calorimeter (Perkin Elmer Diamond DSC) with a heating rate of 10 °C/min under flowing nitrogen condition. The wide-angle X-ray diffraction (WAXD) (model: PAN analytical, EMPYREAN) was measured with a Cu Kα radiation generated at 40 kV and 45 mA.

### Ultra-small angle scattering (USANS) measurement

Pieces about 20 × 20 mm^2^ in size cut from the Cu_50_Zr_50_ ribbons (approximately, 80μm thickness), stacked in three layers to increase the scattering intensity, were attached on a Gadolinium (Gd) diaphragm with a 5/8 inch aperture. The metallic glass ribbons were placed in a SNCM environment, i.e., degassed hydrogenated ethanol (C_2_H_5_OH)/deuterated ethanol (C_2_D_5_OD) mixtures, in the 1 mm path length quartz cell for SNCM experiments. Ethanol was selected due to the low surface tension on the Cu_50_Zr_50_. The H_2_O/D_2_O mixtures with high surface tension were also used to cross-check the results from the ethanol mixtures. The entrapped air bubbles were removed under vacuum.

Ultra-small angle neutron scattering (USANS) was measured with the recently commissioned KIST-USANS at the HANARO cold neutron guide, CG4B, Daejeon, Korea. The KIST-USANS instrument uses a pair of modified Bonse-Hart-Agamalian channel-cut crystals^10,31^ as a monochromator and an analyzer, with a wavelength of 4 Å. The minimum Q-resolution can reach down to ~2 × 10^−5^ Å^−1^. The transmission of Cu_50_Zr_50_ was more than 92%, which assures no multiple scatterings from the metallic samples. Details of multiple scattering is described in the next section. The data reduction was performed with the NIST data reduction package^32^ modified for the KIST-USANS instrument. The USANS intensities are expressed in intensity, I(Q), (unit: counts/sec/Monx10^6^) corrected only with instrumental parameters for the comparison of the scatterings from the sample with a sample environment, or expressed in smeared macroscopic nuclear scattering cross-section, *d*Σ_*sm*_(*Q*)/*dQ*, (unit: cm^−1^sr^−1^) in absolute unit with additional corrections for sample thickness, transmission, and background. The USANS cross-section *d*Σ_*sm*_(*Q*)/*d*Ω in absolute scale were converted to the pin-hole SANS cross-sections *d*Σ(*Q*)/*d*Ω by multiplying a Δ*q*_*V*_/*Q*, where Δ*q*_*V*_ is the vertical divergence of the one-dimensional detector^33^ to estimate the invariant quantity.

### Multiple scattering measurement

**Figure 7:**
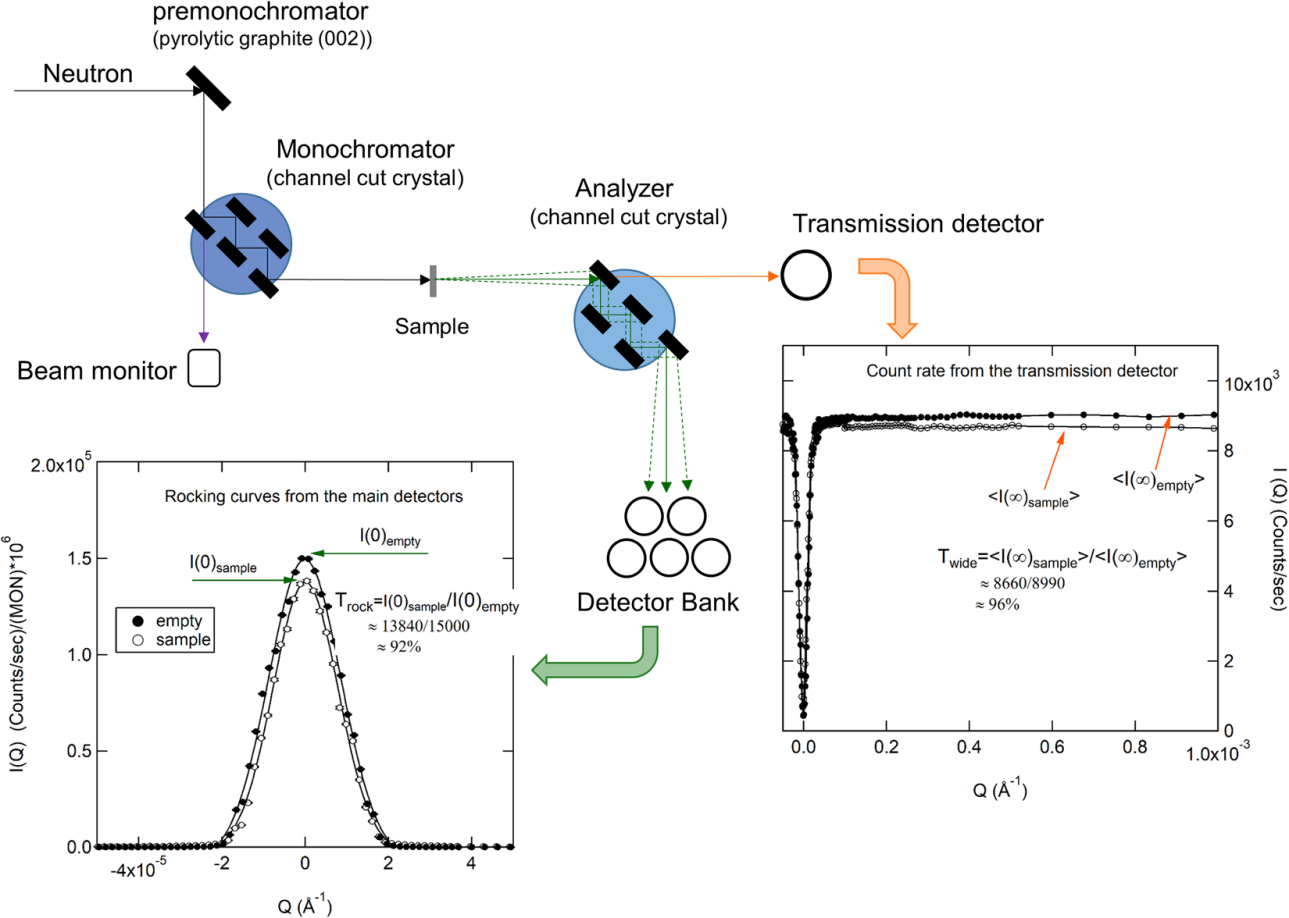
Configuration of the KIST’s ultra-small angle neutron scattering instrument (KIST-USANS), When the monochromator and the analyzer are aligned (Q = 0), the incident neutrons are totally reflected into the main detector bank (rocking peak at Q = 0) and there are no transmitted neutrons on the transmission detector (shown as dip at Q = 0). When the analyzer is rotated by an angle q, only the ultra-small angle scattering corresponding to the same angle q is reflected (see Figs 3–5). When the analyzer is turned further to a large angle (non-Bragg condition), all incident neutrons (except for absorption and incoherent scattering) are transmitted to the transmission detector.

Multiple scattering is proportional to the size of heterogeneity of the scatterer (D) as well as the sample thickness (t), the volume fraction of scatterers (ϕ), the square of neutron wavelength (*λ*^2^), and the contrast (i.e., $\sim {\rm{tD}}{\lambda }^{2}{({\rm{\Delta }}\mathrm{SLD})}^{2}$). Since the USANS measures the microscale heterogeneity, multiple scattering is more concerning than in SANS, which measures on the nano scale. In order to assure that a neutron scatters only once (i.e., no multiple scattering) in the BMG alloys with the large rough surfaces, multiple scattering was estimated by comparing the sample transmissions measured with two independent detectors, a transmission detector and a detector bank (see Fig. 7), in the following manner: after aligning both monochromator and analyzer crystals to satisfy Bragg’s law, the peak intensity of rocking curve is measured at Q = 0 with the detector bank by rotating the analyzer step-by-step through the rocking peak of the main beam. The ratio, ${{\rm{T}}}_{{\rm{rock}}}={\rm{I}}(0{)}_{{\rm{sample}}}/{\rm{I}}{(0)}_{{\rm{empty}}}$, of the rocking peak intensity with and without sample is the sample transmission attenuated by absorption, incoherent scattering, and coherent small angle scattering. A second transmission is measured from the transmission detector at non-Bragg condition. The detector located behind the first analyzer reflector captures all transmitted neutrons not to satisfy the Bragg’s law for the analyzer, which was achieved by rotating the analyzer to a relatively wide angle (Q→∞,afewdegree) from the aligned rocking peak position (Q = 0). The transmission detector measures both the direct beam and forward coherent small angle scattering. The neutron counts collected by the transmission detector are decreased due to absorption and incoherent scattering. Thus, the ratio, T_wide_ = <I(∞)_sample_>/<I(∞)_empty_>, of averaged count rate on the transmission detector with and without the sample represents the sample transmission attenuated by absorption and incoherent scattering.

The ratio ${{\rm{T}}}_{sas}={{\rm{T}}}_{{\rm{rock}}}/{{\rm{T}}}_{{\rm{wide}}}(\,=\exp (\,-\,{\rm{\Sigma }}\cdot t))$ of these two separate transmissions is used to estimate the amount of multiple scattering. If the USANS measurements are free of multiple scattering, T_rock_ ≈ T_wide_. Thus, (1 − T_*sas*_) is the measured scattering probability including multiple scattering. As a rule of thumb, multiple scattering can be neglected when *T*_*sas*_ ≥ 90% or ${\rm{\Sigma }}\cdot t\le 0.1$ where Σ is the macroscopic scattering cross section (i.e., cross section per unit volume) that estimate the attenuation due to small angle scattering and t is the sample thickness^34,35^. If any significant multiple scattering occurs, it can also be shown as a broadening (i.e., flattening) of the sample rocking curve, which can cause an artificial Guinier-like scattering shape. In this study, two independent transmissions were more than 92% for all measurements, ${T}_{sas}\ge 0.95$, and only power law scattering was observed, which confirms that multiple scattering is negligible and the USANS measurements are reliable. Based on this argument, USANS can be measured for homogeneous Cu50Zr50 alloy samples with a thickness ($t={\rm{0.1}}/{\rm{\Sigma }})$ of up to 1.5 mm. If only power law scattering is observed, USANS can also be measured for thicker samples.

## Appendix: Propagation of Uncertainty in the Contrast Matching Plot

The propagation of uncertainty in the two contrast matching methods, the squared root zero angle scattering method and the squared root invariant method, was treated in the following manner;

### A1. Propagation of Uncertainty (vertical error bar) in the zero-angle scattering (or near zero-angle) plot

The measured USANS count (*I*_*SAM*_) of the sample is corrected in absolute scale (unit cm^−1^ sr^−1^), *I*_*cor*_(*Q*), with empty count (or, buffer scattering), (*I*_*EMP*_), sample transmission (*T*_*tran*_), sample thickness (t), detector solid angle (dΩ) and a constant background (*I*_*BKG*_) in the following manner;

A1 $${I}_{cor}(Q)=\frac{d{{\rm{\Sigma }}}_{sm}(Q)}{dQ}=sf\cdot ({I}_{SAM}-{T}_{tran}{I}_{EMP}-(1-{T}_{trans}){I}_{BKG})$$

where sf is an absolute scale factor containing sample transmission (T_wide_) measured in a background region, and a rocking curve peak count of the empty run (pk_EMP_).

A2 $$sf=\frac{1}{{\rm{t}}\cdot d{\rm{\Omega }}\cdot {T}_{wide}\cdot p{k}_{EMP}}$$

The last term, (1 − *T*_*trans*_)*I*_*BKG*_, in Eq. (A1) is a negligibly small constant. Equation (A1) has the following general form

A3 $$f(x)=c(ax\pm bx\pm \,\cdot \,\cdot \,\cdot \,)$$

where a, b, and c are constants. The propagation of uncertainty (*σ*_*f*_) of Eq. (S3) is generally expressed as^36^

A4 $${\sigma }_{f}=\sqrt{{(\frac{\partial f}{\partial x})}^{2}{\sigma }_{x}^{2}+{(\frac{\partial f}{\partial y})}^{2}{\sigma }_{y}^{2}+\ldots }$$

Thus, the propagation of uncertainty of Eq. (S1) is

A5 $${\sigma }_{{I}_{COR}}=\sqrt{{(\frac{\partial {I}_{COR}(Q)}{\partial {I}_{SAM}})}^{2}{\sigma }_{SAM}^{2}+{(\frac{\partial {I}_{COR}(Q)}{\partial {I}_{EMP}})}^{2}{\sigma }_{EMP}^{2}}$$

and

A6 $${\sigma }_{{I}_{COR}}=sf\cdot \sqrt{{\sigma }_{{I}_{SAM}}^{2}+{T}_{trans}{\sigma }_{{I}_{EMP}}^{2}}$$

Equations (A1) and (A6) were used in the USANS data reduction. The measured USANS scattering including the errors is expressed as ${I}_{cor}(Q)\pm {\sigma }_{{I}_{COR}}$.

The USANS total cross-section, ${I}_{cor}(Q)\pm {\sigma }_{{I}_{COR}}$, is the smeared total cross-section due to the one dimensional-detector geometry that has a high resolution only in the horizontal direction. The ${I}_{cor}(Q)\pm {\sigma }_{{I}_{COR}}$ is desmeared with the detector resolution and normalized with Q

A7 $$d{\rm{\Sigma }}(Q)/d{\rm{\Omega }}=({I}_{cor}(Q)\pm {\sigma }_{{I}_{COR}})\cdot {\rm{\Delta }}{q}_{V}/Q$$

which has a standard form of

A8 $$f(x)=a(x\pm b),\,({\rm{where}}\,a={\rm{\Delta }}{q}_{V}/Q,\,{\rm{and}}\,{\rm{b}}={\sigma }_{{I}_{COR}})$$

and because of ∂*f*(*x*)/∂*x* = *a*, the uncertainty is given as

A9 $${\sigma }_{d{\rm{\Sigma }}(Q)/d{\rm{\Omega }}}=\sqrt{{(\frac{{\rm{\Delta }}{q}_{V}}{Q})}^{2}{\sigma }_{d{\rm{\Sigma }}(Q)/d{\rm{\Omega }}}^{2}}$$

which is simply a scaling of the smeared total cross-section, *d*Σ_*sm*_(*Q*)/*dQ*, by Δ*q*_*V*_/*Q*. Δ*q*_*V*_ and Q are the detector divergence and scattering vector, respectively. In the contrast matching plot, where y axis is represented by the squared root of the extrapolated zero angle scattering, $\sqrt{I(Q\to 0)}$, the general form for the uncertainty calculation is given by

A10 $$f(x)=a{x}^{m}$$

and the error is described as Eq. (A4)

A11 $${\sigma }_{f}=\sqrt{{m}^{2}{a}^{2}{x}^{2(m-1)}{\sigma }_{x}^{2}}=m\cdot a\cdot {x}^{(m-1)}{\sigma }_{x}$$

Since $\sqrt{d{\rm{\Sigma }}(0)/d{\rm{\Omega }}}$ is the case of *m* = 1/2 and *a* = 1 in Eq. (A11), the error propagation in $\sqrt{d{\rm{\Sigma }}(0)/\,d{\rm{\Omega }}}$ is expressed as

A12 $${\sigma }_{\sqrt{d{\rm{\Sigma }}(0)/d{\rm{\Omega }}}}=\frac{1}{2}\cdot \frac{\sqrt{d{\rm{\Sigma }}(0)/\,d{\rm{\Omega }}}}{d{\rm{\Sigma }}(0)/\,d{\rm{\Omega }}}{\sigma }_{d{\rm{\Sigma }}(0)/d{\rm{\Omega }}}$$

which is shown in the vertical error bar in Fig. 6(b). Note that in the power law scattering, the $\sqrt{d{\rm{\Sigma }}(0)/\,d{\rm{\Omega }}}$ is replaced with a low Q limit, $\sqrt{I(Q\to 0)}$, for practical purposes. As long as the scattering follows a single power law, a choice of a low Q limit does not affect the matching point.

### Appendix B: Propagation of Uncertainty (vertical error bar) in the squared root invariant plot

The invariant was numerically calculated with the following trapezoidal summation from the measured total scattering section I(Q);

B1 $${Q}_{INV}=\sum _{i=0}^{n}\frac{1}{2}({I}_{i}{Q}_{i}^{2}+{I}_{i+1}{Q}_{i+1}^{2}){\rm{\Delta }}{Q}_{i}$$

where Δ*Q*_*i*_ is defined as Δ*Q*_*i*_ = *Q*_*i* + 1_−*Q*_*i*_, instead of ${\rm{\Delta }}{Q}_{i}=({Q}_{i+1}-{Q}_{i})/n$, since the USANS intensity was measured with different intervals of Q for each angular region. For simplicity in the notation *I*_*i*_ is used for representing ${I(Q)}_{{\rm{i}}}\,(\,\approx \,{d{\rm{\Sigma }}(Q)}_{{\rm{i}}}/d{\rm{\Omega }})$. In order to separate out the 1^st^ (*i* = 0), the last (*i* = *n*) term, and the rest term ($i=1\sim n-1$) for numerical calculation, Eq. (B1) was expanded and rearranged as

B2 $${Q}_{INV}=\frac{{\rm{1}}}{{\rm{2}}}{I}_{0}{Q}_{0}^{2}({Q}_{1}-{Q}_{0})+\frac{{\rm{1}}}{{\rm{2}}}\sum _{i=1}^{n-1}{I}_{i}{Q}_{i}^{2}({Q}_{i+1}-{Q}_{i-1})+\frac{{\rm{1}}}{{\rm{2}}}{I}_{n}{Q}_{n}^{2}({Q}_{n}-{Q}_{n-1})$$

Each of the partial derivatives of Eq. (B2) are

B3 $$\frac{\partial {Q}_{INV}}{\partial {I}_{0}}=\frac{1}{2}{I}_{0}^{2}({Q}_{1}-{Q}_{0}),\frac{\partial {Q}_{INV}}{\partial {I}_{i}}=\frac{1}{2}\sum _{i=1}^{n-1}{Q}_{i}^{2}({Q}_{i+1}-{Q}_{i-1})\frac{\partial {Q}_{INV}}{\partial {I}_{n}}=\frac{1}{2}{Q}_{n}^{2}({Q}_{n}-{Q}_{n-1})$$

By inserting Eq. (B3) into Eq. (A4), we obtain the following expression for the propagation of uncertainty in invariant

B4 $${\sigma }_{{Q}_{inv}}=\sqrt{{(\tfrac{1}{2}{Q}_{0}^{2}({Q}_{1}-{Q}_{o}){\sigma }_{{I}_{0}})}^{2}+{(\tfrac{1}{2}\sum _{i=1}^{n-1}{Q}_{i}^{2}({Q}_{i+1}-{Q}_{i-1}){\sigma }_{{I}_{i}})}^{2}+{(\tfrac{1}{2}{Q}_{n}^{2}({Q}_{n}-{Q}_{n-1}){\sigma }_{{I}_{n}})}^{2}}$$

The invariant including the error propagation is expressed as ${Q}_{inv}\pm {\sigma }_{{Q}_{inv}}$.

In the contrast matching plot where the y axis is $\sqrt{{Q}_{inv}}$, the vertical error bar is given by

B5 $${\sigma }_{\sqrt{{Q}_{inv}}}=\frac{1}{2}\cdot \frac{\sqrt{{Q}_{inv}}}{{Q}_{inv}}{\sigma }_{{Q}_{inv}}$$

Note that in Fig. 6(a) (in the main text), the vertical error bars within the symbols. Apparently, the squared root invariant matching method shows smaller error bars than the squared root of the extrapolated zero angle scattering, $\sqrt{d{\rm{\Sigma }}(0)/d{\rm{\Omega }}}$, method. However, regardless of the method, the densities estimated from both methods are identical within experimental error.

## Electronic supplementary material

Supplementary Information
