# Supplementary material for: Structural homogeneity and mass density of bulk metallic glasses revealed by their rough surfaces and ultra-small angle neutron scattering (USANS)
Source: Sci Rep. 2018 Aug 28;8:12986. doi: 10.1038/s41598-018-30333-9 (PMC6113253; doi:10.1038/s41598-018-30333-9)
Supplement: Supplementary file 1 — Supplementary Information [file 41598_2018_30333_MOESM1_ESM.docx]

**(Supplementary Information)**

Structural homogeneity and mass density of bulk metallic glasses revealed by their rough surfaces and ultra-small angle neutron scattering (USANS)

Man-Ho Kim*^a^**, Jin-Yoo Suh*^b^*, Eric Fleury*^c^*, Su Gyeong Han*^a^*, Kyung Tae Hong^d^

*^a^ Advanced Analysis Center, Korea Institute of Science and Technology, Seoul 02792, Republic of Korea*

*^b^ High Temperature Energy Materials Research Center, Korea Institute of Science and Technology, Seoul 02792, Republic of Korea*

*^c^ LEM3, UMR CNRS 7239, Université de Lorraine, 57000 Metz, France*

*^d^* Center for Materials Architecturing, Korea Institute of Science and Technology, Seoul, 02792, *Republic of Korea*

^*^Corresponding author: [man-hokim@kist.re.kr](mailto:man-hokim@kist.re.kr)

**Preliminary USANS and SANS data.**

USANS and SANS measurements were performed for the three bulk metallic ribbons.; as-cast, sub-Tg annealed, and crystallized samples, are shown in Fig S1. As confirmed in DSC thermal analysis and XRD patterns, the inner structures of as-cast and sub-Tg annealed samples are homogeneous in the atomic level. These two homogeneous samples must show no small angle neutron scattering. However, unlike our expectation, the homogeneous samples show the strong power law scattering in USANS and SANS measurements (Fig. S1). The heterogeneous (i.e., crystallized) bulk metallic ribbons show a clear knee (i.e., Guinier scattering), as expected, around $Q\sim10^{-3}$Å^-1^ and power law scattering at the USANS region. The origin of the power law scatterings in both the homogeneous and the heterogeneous BMG ribbons is investigated in this study (see main text) and used to identify the inner homogeneity and to determine the mass density.

**
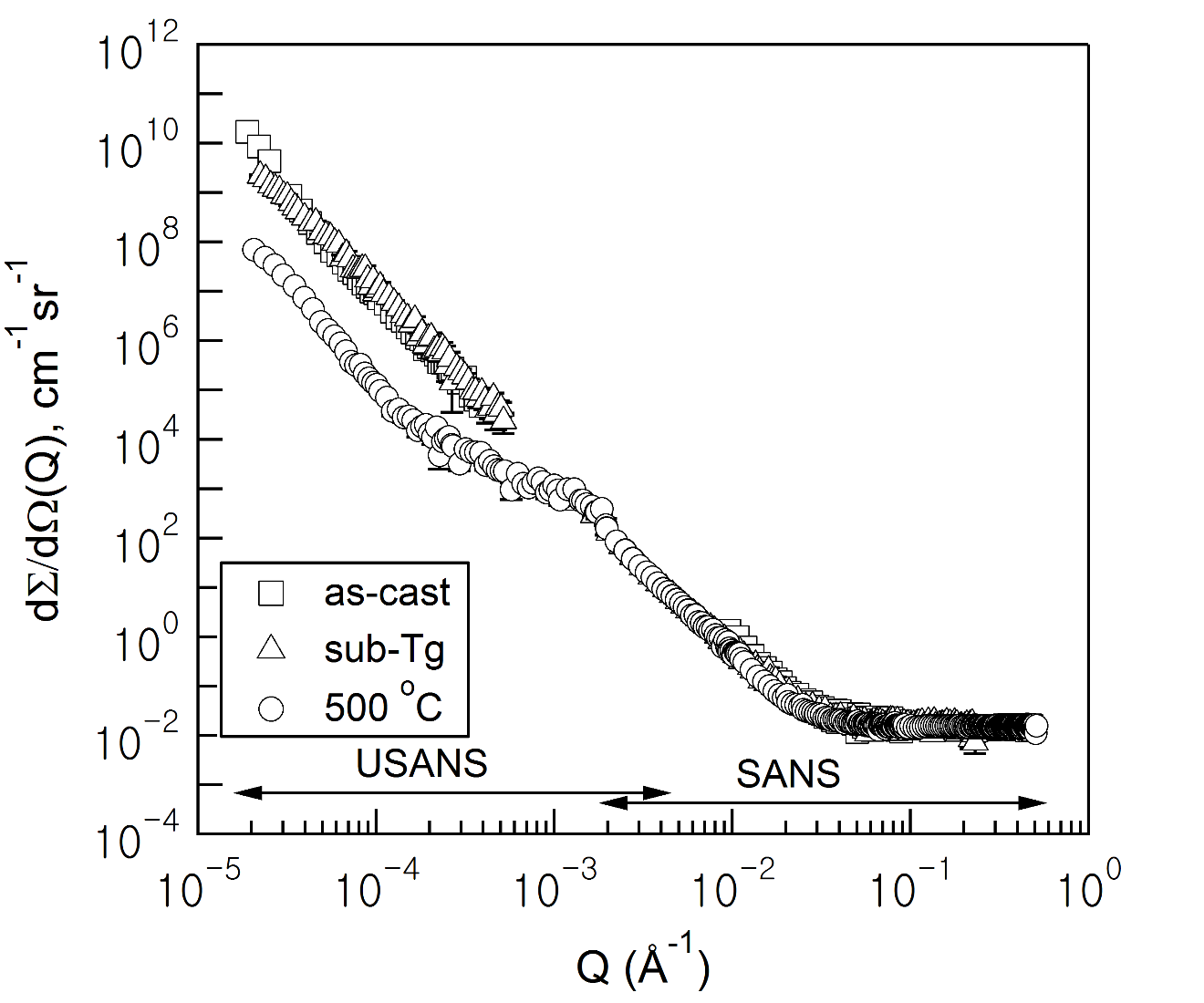
**

**Figure S1.** Preliminary USANS-SANS data of as-cast Cu_50_Zr_50_ BMG and annealed samples. USANS scattering was desmeared to match with the pinhole geometry of SANS. The as-received samples (as-cast, open square) were lightly annealed at 400 °C (for 1hr) below the glass transition temperature Tg (sub-Tg, open triangle), and were isothermally crystallized at 500 °C (open circle).

The gap between USANS and SANS in the as-cast and sub-Tg annealed samples is due to the USANS scattering reaching the empty scattering at beyond Q~ 5x 10^-4^ Å^-1^ while the SANS scattering reached the resolution limit.


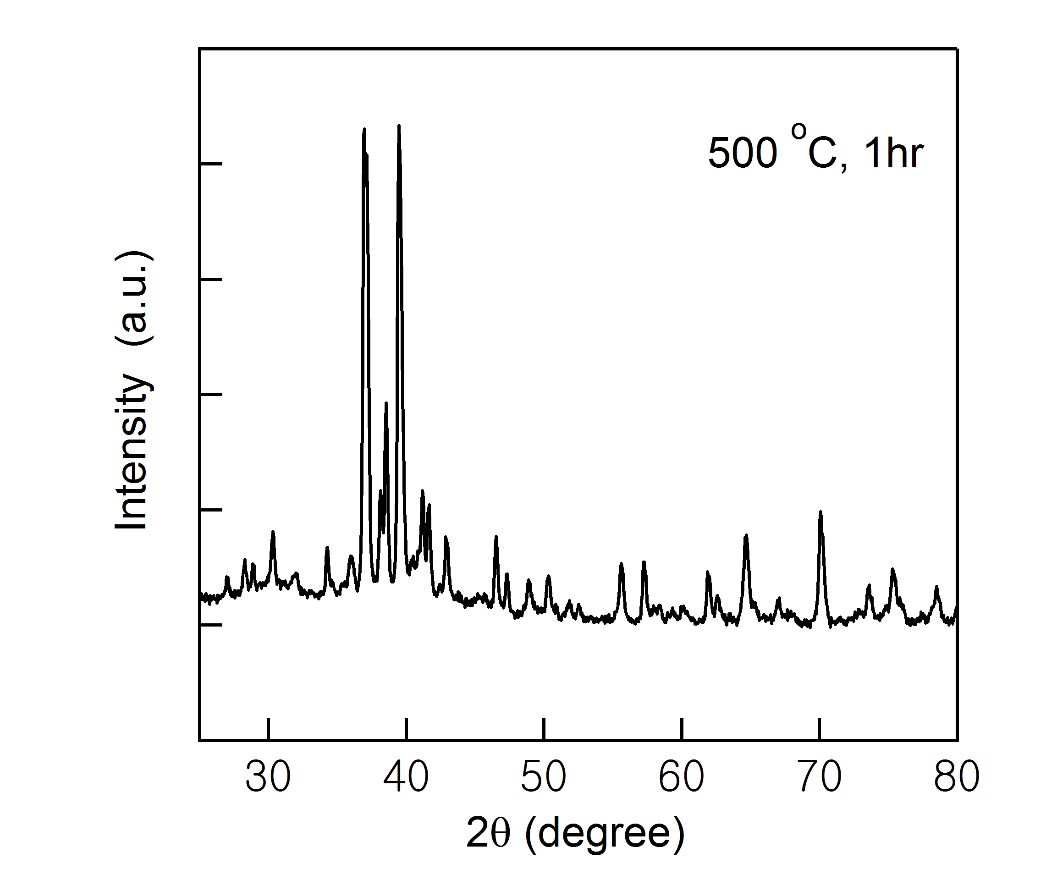


**Figure S2.** XRD pattern of Cu_50_Zr_50_ BMG crystallized at 500 ^o^C for 1hr.
